# An Efficient Finger Vein Recognition Method Based on Improved Lightweight MobileNet

**DOI:** 10.3390/s26051634

**Published:** 2026-03-05

**Authors:** Xuhui Zhang, Yuxi Liu, Yixin Yan, Jiabin Li, Lei Xu

**Affiliations:** 1 School of Measurement and Control Technology and Communication Engineering, Harbin University of Science and Technology, Harbin 150080, China; zxh_99ok@163.com (X.Z.); 2320610180@stu.hrbust.edu.cn (Y.L.); 2420610179@stu.hrbust.edu.cn (L.X.); 2 School of Electrical, Electronic and Intelligent Engineering/Department of Electrical Engineering, Harbin Cambridge University, Harbin 150069, China; ljb0709@163.com

**Keywords:** finger vein recognition, lightweight neural networks, deep learning, image preprocessing, efficient identification

## Abstract

**Highlights:**

**What are the main findings?**

- The proposed LCNN framework significantly enriches finger vein feature representation while minimizing structural complexity and computational burden without impairing recognition precision.
- A robust multi-stage preprocessing strategy combined with a compact network design achieves stable identification under illumination variation and resource-limited conditions.

**What are the implications of the main findings?**

- The results affirm that lightweight deep architecture preserves superior accuracy while enabling efficient real-time integration within embedded biometric recognition systems.
- The proposed method provides useful design insights for developing efficient, scalable, and secure biometric recognition systems in future edge-intelligent applications.

**Abstract:**

Finger vein recognition has emerged as a highly robust and intrinsically stable biometric technology, demonstrating great potential in identity authentication and intelligent security applications. However, conventional methods still suffer from constraints in feature representation and computational efficiency, particularly under challenging conditions such as illumination variation, pose deviation, and noise interference. To address these challenges, this study presents an efficient finger vein recognition approach based on a lightweight convolutional neural network (LCNN) architecture. The proposed framework integrates a multi-stage image preprocessing pipeline for automatic vein region detection, advanced denoising, and refined texture enhancement, which is subsequently followed by compact feature modeling within a lightweight deep network. Extensive experiments on the public Shandong University Machine Learning and Applications-Homologous Multi-Modal Traits (SDUMLA-HMT) dataset and a self-acquired Laboratory Finger-Vein (Lab-Vein) dataset validate the superiority of the proposed method, achieving recognition accuracies of 97.1% and 98.3%, respectively, surpassing existing benchmark models. Moreover, the model demonstrates notable reductions in parameter complexity and computational cost, achieving an average inference time of only 12.6 ms, which confirms its strong real-time capability and suitability for embedded deployment. Overall, the proposed approach attains a desirable trade-off between accuracy and efficiency, offering meaningful implications for the advancement of lightweight biometric recognition systems.

## 1. Introduction

Information security has become an increasingly critical concern, driving extensive research into biometric-based identification technologies over the past decade. Among them, finger vein recognition has gained prominence as a compelling biometric authentication modality owing to its intrinsic merits, including robust liveness detection, high resistance to forgery, and enhanced privacy preservation [1,2]. Nevertheless, finger vein images typically exhibit subtle textural features and inherently low contrast, rendering them highly vulnerable to variations in illumination and finger posture. These factors present substantial challenges to the robustness and reliability of feature extraction and matching procedures [3,4]. Early investigations into finger vein recognition predominantly depended on conventional image processing techniques and handcrafted feature extraction methods, including maximum curvature, repeated line tracking, and Gabor filtering. Although these methods possess relatively simple architectures, they are highly sensitive to variations in imaging conditions and exhibit limited robustness. With the rapid progress of deep learning, convolutional neural networks have been introduced into finger vein recognition, facilitating the automatic extraction of multi-level vein texture features and substantially enhancing recognition performance. Nonetheless, such models frequently involve deep architecture, large parameter volumes, and considerable computational costs, which hinder their applicability in real-time scenarios on mobile or embedded platforms. To achieve a balance between performance and efficiency, recent studies have increasingly emphasized lightweight network design. These compact architectures employ depthwise separable convolutions, feature compression, and attention mechanisms to effectively reduce the computational burden while preserving competitive accuracy [5,6]. Despite the promising performance of lightweight networks in generic image recognition tasks, their direct application to finger vein recognition remains challenging due to insufficient feature representation capacity and the imbalanced modeling of local and global texture information [7,8,9].

Motivated by these limitations, this work presents a lightweight convolutional neural network (LCNN)-based finger vein recognition framework designed to achieve an optimal balance between computational efficiency and recognition accuracy. The proposed method effectively exploits deep representations of vein textures under limited computational budgets, enhancing its deployability and recognition robustness in resource-constrained environments.

## 2. Related Work

Accompanied by the rapid advancement of deep learning and attention mechanisms, research on finger vein recognition has gradually evolved from traditional handcrafted algorithms toward end-to-end feature learning paradigms. Recent investigations have primarily focused on three mainstream directions: the refinement of network architectures, the integration of attention mechanisms, and the design of lightweight models. These endeavors aim to achieve an optimal trade-off between recognition accuracy and computational efficiency, particularly under resource-constrained conditions [10,11].

(1) Feature Modeling Based on Attention Mechanisms and Transformer Architectures

Recent developments have explored the integration of attention mechanisms and Transformer-based architecture to enhance feature modeling for finger vein recognition. Li et al. [12] proposed a hybrid model, ViT-Cap, combining Vision Transformer and Capsule Network to leverage both global context modeling and local spatial representation. This approach improves robustness while maintaining high recognition accuracy. However, its complex design, high computational cost, and sensitivity to input size hinder practical deployment. To balance accuracy and efficiency, Huang et al. [13] introduced ALA-Net, which utilizes axial attention and grouped convolution to effectively capture long-range vein structures and local textures. Although ALA-Net achieves a good trade-off between precision and model compactness, its generalization under limited training data remains limited. Ke et al. [14] proposed GLA-FD, a Vision Transformer incorporating feature decoupling and a hierarchical attention module to improve robustness against variations in illumination and finger displacement. While it performs well on public datasets, the inherent complexity of Transformer-based models continues to challenge real-time deployment.

(2) Efficient Recognition Methods Based on Lightweight Convolutional Architectures

In response to the growing demand for model simplification and embedded deployment in finger vein recognition, numerous lightweight convolutional architectures have been proposed. Tahir et al. [15] introduced FV-EffResNet, employing depthwise separable and pointwise convolutions to streamline feature extraction. With the integration of the Swish activation function and a cyclical learning rate strategy, the model achieves high accuracy with fewer parameters, though it underperforms in open-set and multi-task scenarios. Tran et al. [16] proposed VeinKAN, combining the Kolmogorov–Arnold Network (KAN) with Inception V3 to enhance multi-scale feature learning. However, its substantial parameter size hinders deployment on resource-constrained devices. To improve robustness, Li et al. [17] developed BMFAN, a dual-branch network integrating local and global attention mechanisms. While effective, its complex architecture introduces significant computational overhead. Nadir et al. [18] combined MobileNetV2 with SVM to create a hybrid model balancing accuracy and efficiency, although it remains sensitive to illumination and image quality variations. Deshmukh et al. [19] enhanced MobileNetV3 with feature optimization and depthwise convolutions, yet its generalization to diverse conditions is limited.

Additionally, several studies have addressed model functional enhancement and image preprocessing. Das et al. [20] proposed a CNN-based method demonstrating robustness under varying image qualities, but with increased structural complexity and training time. Kotwal et al. [21] utilized a ResFPN-based enhancement strategy to improve contrast and accuracy, although it relies heavily on annotated data. Shen et al. [22] presented a lightweight model combining Mini-RoI extraction, curvature optimization, and triplet loss, enabling high accuracy and incremental class adaptation, yet its cross-database generalization remains insufficient.

Despite notable progress in finger vein recognition, several key challenges persist. Transformer-based and attention-enhanced models excel at feature modeling but suffer from high complexity and parameter redundancy, which hinder deployment on resource-constrained devices. In contrast, lightweight networks improve efficiency yet often compromise texture richness, while inadequate multi-scale fusion and limited local-global representation reduce robustness under diverse imaging conditions. Therefore, this study aims to enhance fine-grained texture encoding and design a compact yet effective framework that balances accuracy and efficiency.

## 3. Finger Vein Image Preprocessing Method

Finger vein images are easily influenced by illumination changes, posture variations, and noise, leading to reduced deep network performance. This study proposes a multi-stage preprocessing pipeline comprising ROI localization, enhancement and denoising, and vein segmentation to obtain stable and consistent vein features for recognition tasks.

### 3.1. ROI Localization

Accurate extraction of the finger pad region (ROI), which contains essential vein texture information, is vital for reliable feature extraction [23,24]. An automated ROI extraction algorithm based on adaptive thresholding and morphological operations is employed. The finger vein image is converted to grayscale and enhanced using Contrast Limited Adaptive Histogram Equalization (CLAHE) to improve contrast and suppress noise.

(1) $$I_{eq}\left(x,y\right)=T_{CLAHE}\left(I\left(x,y\right)\right)$$

The enhanced image is binarized using adaptive thresholding based on the local intensity distribution. Morphological operations (hole filling, opening, and closing) are then applied to denoise and smooth the edges. Finally, the largest connected component corresponding to the finger region is extracted using the regionprops function, and its bounding box is refined to exclude the palm and fingertip areas, as shown in Equation (2).

(2) $$R_{new}=\left[x+0.1w,y+0.1h,0.8w,0.8h\right]$$

**Figure 1:**
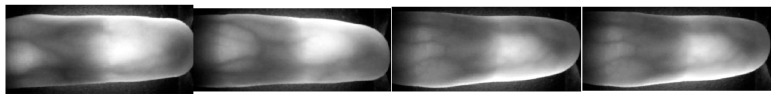
Original finger vein image.

**Figure 2:**
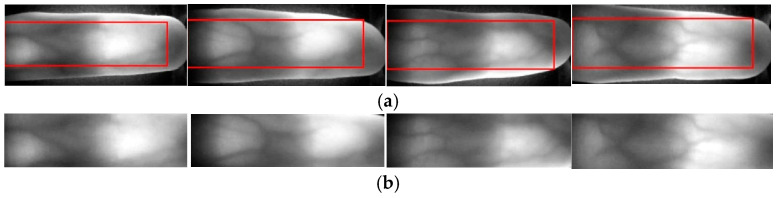
ROI localization results for four finger vein samples acquired under different lighting and vein patterns: (**a**) ROI annotation showing the automatically detected regions of interest; (**b**) final extracted ROI regions after cropping corresponding to the annotated areas.

The final ROI is obtained after this cropping step, which ensures spatial consistency and reduces irrelevant background. Figure 1 and Figure 2 illustrate the original finger vein image and the ROI localization process, respectively.

### 3.2. Image Enhancement and Denoising

After ROI extraction, uneven illumination and noise may still obscure vein patterns [25]. Traditional Gabor-based enhancement approaches have also been widely used in finger and palm vein recognition to emphasize multi-directional texture details; however, these handcrafted filter-based methods are often sensitive to illumination variations [26]. To enhance visibility more robustly, this study applies a multi-stage adaptive enhancement and denoising method. CLAHE is first used to improve local contrast, as defined in Equation (3).

(3) $$I_{enh}\left(x,y\right)=T_{clahe}\left(I_{roi}\left(x,y\right)\right)$$

Finally, a linear fusion strategy combines the denoised and original images to enhance readability without over-brightening, as given in Equation (4).

(4) $$I_{enh}=\alpha I+\beta I_{wavelet}$$

**Figure 3:**
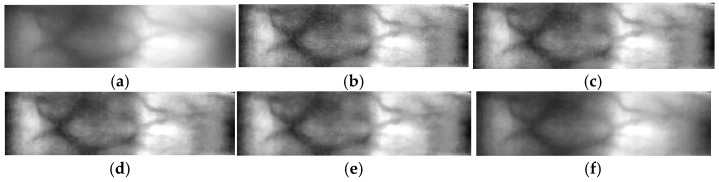
Illustration of the finger vein image enhancement and denoising process. Step-by-step results of the enhancement and denoising process: (**a**) original ROI; (**b**) contrast enhancement using CLAHE; (**c**) after median filtering; (**d**) after Gaussian filtering; (**e**) after wavelet-based denoising with soft thresholding; (**f**) final enhanced result after linear fusion.

Through comprehensive experimental evaluation, the parameter setting α=0.7 and β=0.3 achieves the best balance between natural brightness preservation and maximum enhancement of vein visibility. As illustrated in Figure 3, the enhancement and denoising process significantly accentuates vein contours while diminishing background interference.

### 3.3. Vein Segmentation and Skeletonization

The enhanced image is further post-processed using a two-stage procedure, which includes the Otsu thresholding method followed by lookup-table-based skeletonization to obtain a clear and continuous vein structure.

(1) Vein Segmentation

The Otsu thresholding algorithm automatically selects an optimal threshold $T^{*}$ by maximizing the variance between foreground and background classes [27]. The mathematical expression is provided in Equation (5).

(5) $$\sigma_{b}^{2}=\omega_{1}{\left(m_{1}-m_{g}\right)}^{2}+\omega_{2}{\left(m_{2}-m_{g}\right)}^{2}$$

**Figure 4:**
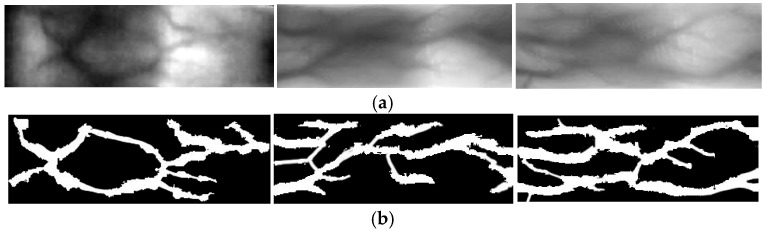
Binarization results before and after processing for three different finger vein samples: (**a**) before processing; (**b**) after processing.

The segmentation results before and after binarization are presented in Figure 4, which demonstrates the algorithm’s ability to partition vein and background regions adaptively.

(2) Vein Skeletonization

**Figure 5:**
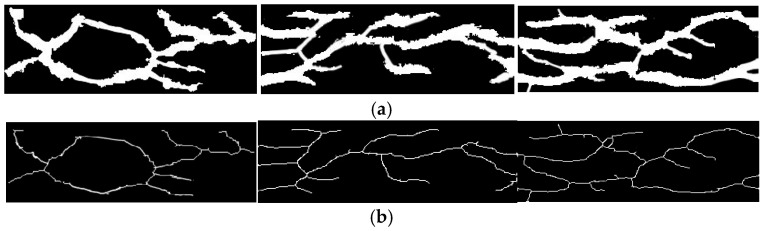
Vein skeletonization results before and after processing for multiple finger vein samples: (**a**) before processing (binarized images); (**b**) after skeletonization.

Due to variations in blood flow and temperature, the width of finger veins may fluctuate. However, their skeleton structure remains relatively stable. To extract this stable centerline representation, an 8-neighborhood pixel thinning algorithm based on a lookup table (LUT) is employed in this study. The weighted sum of the center pixel’s neighborhood is computed according to Equation (6). The final skeletonization result is shown in Figure 5.

(6) $$V=\sum_{i=1}^{8}p_{i}\times w_{i}$$

The proposed method effectively removes redundant information while preserving the connectivity and topology of the vascular structure. The structuring element was set to 3 × 3 based on the average vein width, and segmentation performance remained stable when this parameter varied slightly (2 × 2 to 5 × 5). Through the proposed multi-stage preprocessing pipeline, high-quality vein patterns are automatically extracted and enhanced, providing a robust foundation for subsequent feature extraction and recognition.

(3) Comparison with DL-Based Vein Segmentation

Two representative deep learning (DL) models, U-Net and MobileNet-U-Net, were evaluated on the same finger-vein dataset under identical conditions. As shown in Table 1, both achieved slightly higher Dice and IoU values but required substantially more computation and parameters. The proposed Otsu-based method attains comparable accuracy with markedly lower complexity and faster inference, making it more suitable for lightweight real-time applications.

## 4. Lightweight Finger Vein Recognition Algorithm Based on CNN

### 4.1. Design Principles of the Lightweight Convolutional Neural Network

This study tackles the issues of large parameter size, high computational cost, and inefficiency of traditional CNNs on embedded platforms by proposing a task-oriented Lightweight Convolutional Neural Network (LCNN) optimized for finger vein recognition. Inspired by lightweight designs like inverted residual blocks, channel shuffle, and SE attention, LCNN innovatively integrates and restructures them through modular co-design, achieving an adaptive trade-off between representational power and computational efficiency under low-contrast, fine-grained imagery.

(1) Inverted Residual Structure with Linear Bottleneck

**Figure 6:**
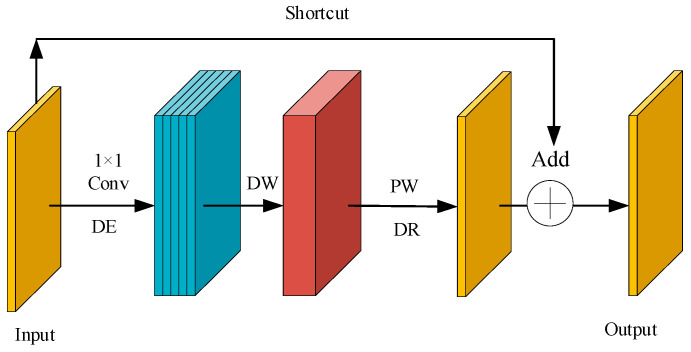
Linear bottleneck inverted residual structure.

MobileNetV2 [28] employs an inverted residual structure with a linear bottleneck, reducing parameters and FLOPs while preserving discriminative features. Unlike ResNet’s “compress-expand” strategy, it follows an “expand-compress” paradigm: a 1 × 1 pointwise convolution enlarges the feature dimension, depthwise convolution extracts spatial information, and another 1 × 1 projection restores compactness. LCNN reconfigures this structure as a lightweight expansion unit tuned to shallow spatial scales and delicate vascular patterns of finger-vein imagery, achieving efficient representation with minimal computation, as illustrated in Figure 6.

(2) Channel Shuffle Operation

**Figure 7:**
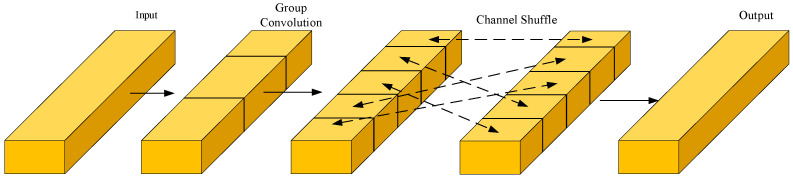
Channel shuffle operation in ShuffleNet.

Although group convolution reduces computational complexity, it limits information exchange among channel groups. ShuffleNet mitigates this by introducing channel shuffle, which enables cross-group communication through feature map permutation. In LCNN, this mechanism is incorporated into the dual-branch Block 2, enhancing cross-channel fusion of fine-grained vein textures. Such customized integration enables later convolutions to capture both local and global patterns more effectively without extra cost. The overall process is illustrated in Figure 7.

(3) SE Channel Attention Mechanism

**Figure 8:**
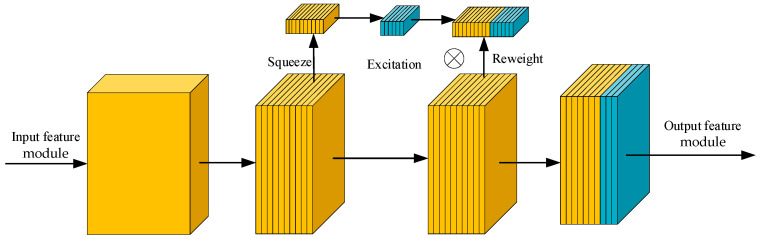
SE Channel attention module.

The SE module enhances channel-wise representations through squeeze, excitation, and recalibration. As shown in Figure 8, global average pooling first produces channel descriptors, which pass through a compact two-layer fully connected network to learn inter-channel dependencies. In LCNN, a reduced bottleneck ratio and lightweight design allow the SE module to emphasize subtle vascular contrasts while suppressing redundancy for discriminative efficiency.

(4) Lightweight Activation Function: h-swish6

LCNN adopts the h-swish activation function proposed in MobileNetV3 to balance nonlinear representational capacity and computational efficiency. Compared with the conventional ReLU6 function, h-swish provides a smoother transition over the interval [−3,3], thereby mitigating the inactive-neuron issue associated with ReLU while approximating sigmoid-like behavior at low computational cost. Accordingly, h-swish is selected as the default activation function throughout LCNN to further enhance learning performance on embedded devices.

### 4.2. Network Architecture and Parameter Settings

Building on the aforementioned design principles, the proposed LCNN features an enhanced modular architecture tailored to the specific characteristics of finger-vein images. Rather than introducing new operators, its contribution lies in integrating and reconfiguring lightweight mechanisms into a coherent, task-adaptive hierarchy, achieving a favorable trade-off between texture-feature fidelity and computational cost.

(1) Overall Network Architecture Design

**Figure 9:**
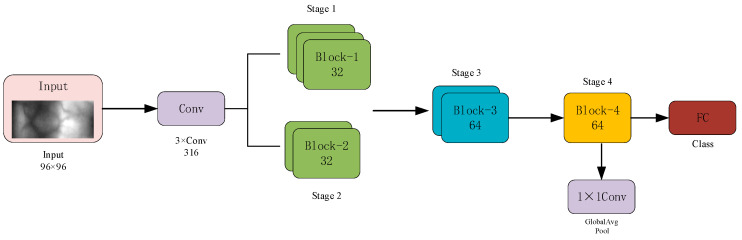
Overall architecture of the proposed LCNN.

The proposed LCNN employs a multi-stage hierarchical architecture comprising an initial convolutional layer, a backbone feature extractor, and a classification head. The input first undergoes a 3 × 3 convolution and max pooling for initial down-sampling, then passes through four progressive stages that abstract features from shallow textures to deep semantics. Early layers capture edges and fine-grained details, while middle and higher layers encode semantic and structural information. This hierarchical design promotes a gradual transition from local vascular patterns to holistic vein structures, which is crucial for low-contrast vein recognition. Finally, a 1 × 1 convolution, global average pooling, and a fully connected layer produce the classification output, as shown in Figure 9.

Each stage of the proposed LCNN is built upon depthwise separable convolution (DWConv) as the core operation. To further enhance feature interaction and channel selection capability, the architecture integrates both the Squeeze-and-Excitation attention mechanism and the channel shuffle strategy. These enhancements enable the network to maintain strong feature representation performance under lightweight constraints.

(2) Design of Four Types of Lightweight Convolutional Modules

**Figure 10:**
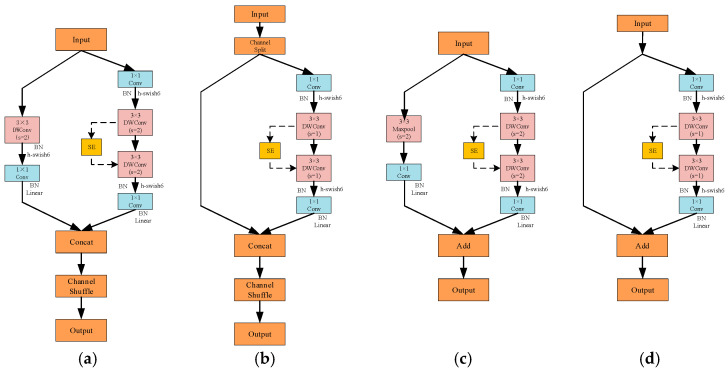
Structures of the Four Lightweight Convolutional Blocks in the Proposed LCNN. (**a**) block-1; (**b**) block-2; (**c**) block-3; (**d**) block-4.

LCNN enhances architectural flexibility and efficiency by reorganizing conventional lightweight operations into four task-specific convolutional modules. Rather than being direct copies of existing units, each block is carefully customized to fulfill a distinct functional objective while adhering to the overall lightweight design constraint, as illustrated in Figure 10.

① Block-1: This module employs a depthwise-separable convolution with a stride of 2 for spatial downsampling and integrates a SE attention mechanism to enhance channel-wise feature recalibration, effectively reducing the spatial resolution while strengthening salient feature representations.

② Block-2: Block-2 adopts a dual-branch convolutional structure combined with a channel shuffle mechanism to enable cross-group information exchange. This design enhances feature fusion across different channel groups while maintaining computational efficiency.

③ Block-3: This module integrates a convolutional branch and a pooling branch in parallel, enabling multi-scale feature enhancement through branch-wise fusion while effectively capturing both local details and global context.

④ Block-4: Positioned near the output stage, Block-4 performs feature compensation to improve the stability and discriminative power of deep representations, functioning as a refinement unit that strengthens final high-level features before classification.

(3) Parameter Configuration of Each Network Stage

The main parameter settings of the proposed network are summarized in Table 2. The overall design follows the principle of progressively increasing the number of channels layer by layer, while controlling computational complexity to maintain efficiency. Across the four feature abstraction stages, the output channel dimensions are gradually expanded to enrich the feature space. Finally, a 1 × 1 convolution is applied to aggregate features into a compact 128-dimensional representation for classification.

(4) Architectural Strengths and Advantages

Although depthwise separable convolution, channel shuffle, and SE attention have been widely applied in previous lightweight networks, their task-driven integration and hierarchical reorganization in LCNN represent the key innovation of this work. The four modular blocks are co-designed to provide complementary benefits-efficient spatial abstraction, cross-channel interaction, and adaptive multi-scale enhancement. Unlike generic mobile backbones, LCNN is customized for finger-vein imagery, maintaining robust feature extraction under low-contrast conditions. Consequently, it achieves about 50% reductions in parameters and FLOPs while preserving high recognition accuracy and efficiency on resource-limited platforms.

## 5. Experimental Design and Result Analysis

### 5.1. Experimental Environment and Datasets

All model training and testing procedures in this study were conducted on a host computing platform. The experimental system was configured with an Intel Core i7-14650HX @ 2.20 GHz processor, an NVIDIA GeForce RTX 4060 GPU, and 32 GB of RAM, running on the Windows 11 operating system. The software environment was based on Python 3.8 and implemented using the PyTorch 2.2.1 deep learning framework, with CUDA 11.1 and cuDNN 8.1 providing GPU acceleration. The detailed system configuration is summarized in Table 3.

The proposed LCNN model was evaluated on both the public SDUMLA-HMT dataset and a self-constructed dataset (Lab-Vein) to assess its generalization capability and practical application performance.

(1) SDUMLA-HMT Dataset

The SDUMLA-HMT dataset was developed by the Machine Learning and Data Mining Laboratory of Shandong University. It comprises finger vein images collected from 106 volunteers. For each participant, samples were captured from six fingers-namely, the index, middle, and ring fingers of both hands-resulting in a diverse and comprehensive finger vein image set. The acquisition conditions, including illumination, finger posture, and image resolution, were maintained consistently, making the dataset a widely adopted benchmark for finger vein recognition research. In this study, each finger of each subject is regarded as an independent class, thereby formulating a multi-class classification problem for evaluation.

(2) Self-Constructed Lab-Vein Dataset

In order to ensure data consistency and controllability, a self-constructed Lab-Vein finger vein dataset was built under controlled laboratory conditions. A total of 20 volunteers participated in the data collection process. For each subject, 10 fingers were captured, and 10 images per finger were acquired under varying finger postures and pressing forces, resulting in 2000 raw images. The acquisition was performed at a room temperature of 25 °C, using an 850 nm near-infrared (NIR) light source and a camera with a resolution of 640 × 480 pixels. To augment data diversity and improve model generalization, various image enhancement techniques were applied, including gamma correction, cropping, translation, and rotation. As a result, the dataset was expanded to approximately 8000 samples.

(3) Data Partitioning Strategy

A subject-disjoint partitioning strategy was employed in all experiments to ensure fairness and reproducibility, guaranteeing that samples from the same subject never appeared in both the training and testing sets. This strategy prevents identity leakage and provides a more realistic evaluation of the model’s generalization ability. Both the SDUMLA-HMT and Lab-Vein datasets were partitioned with an 8:2 ratio for training and testing, respectively. This configuration guarantees that the training set includes a sufficient number of samples for effective model learning, while the testing set remains strictly independent, thereby enabling robust and unbiased performance evaluation.

### 5.2. Hyperparameter Settings and Evaluation Metrics

All experiments were conducted using a uniform set of training hyperparameters, ensuring a fair comparison between different network architectures under consistent conditions. The input image size was fixed at 128 × 128 pixels to balance finger vein texture fidelity and computational efficiency. The Adam optimizer was employed with an initial learning rate of 0.001, which provided stable convergence throughout the training process. The batch size was set to 100, and the number of training epochs was fixed at 200 to ensure sufficient learning iterations. All models were optimized using the cross-entropy loss function, which is well-suited for multi-class finger vein recognition tasks. This unified training configuration provided a fair and reproducible foundation for performance comparisons between classical CNNs and the proposed lightweight LCNN variants.

To comprehensively assess the performance of the proposed model, the following evaluation metrics were employed: Accuracy, Precision, Recall (Sensitivity), F1-Score, and Loss. Among them, accuracy and loss were primarily used during model training and for comparative performance analysis across different architectures. The additional metrics—precision, recall, and F1-score-were applied in the Lab-Vein dataset experiments as supplementary indicators to enhance the completeness and robustness of the evaluation.

(1) Accuracy

Accuracy is used to measure the overall proportion of correctly classified samples and serves as one of the most commonly used performance metrics for recognition tasks, as defined in Equation (7).

(7) $$A\mathrm{ccuracy}=\frac{TP+TN}{TP+TN+FP+FN}$$

(2) Precision

Precision is used to measure the accuracy of positive predictions among all predicted positive samples. Its formula is shown in Equation (8).

(8) $$Precision=\frac{TP}{TP+FP}$$

(3) Sensitivity

Sensitivity measures the model’s ability to correctly identify actual positive samples. The specific formula is given in Equation (9).

(9) $$Sensitivity=\frac{TP}{TP+FN}$$

(4) F1-Score

F1-Score is the harmonic means of precision and sensitivity, providing a balanced measure of the model’s accuracy and recall ability. The formula is given in Equation (10).

(10) $$F1=\frac{2\times Precision\times Sensitivity}{Precision+Sensitivity}$$

### 5.3. Experimental Results and Analysis

#### 5.3.1. Results on the Public Dataset SDUMLA-HMT

To evaluate the recognition performance of the proposed LCNN model on the public dataset, four representative finger vein recognition networks were selected as comparison baselines: ViT-Cap [12], ALANet [13], FV-EffResNet [15], and VeinKAN [16]. These models have demonstrated strong feature extraction capabilities and high recognition accuracy in their original studies. Therefore, their performance serves as a reliable benchmark for assessing the effectiveness of the proposed method.

**Figure 11:**
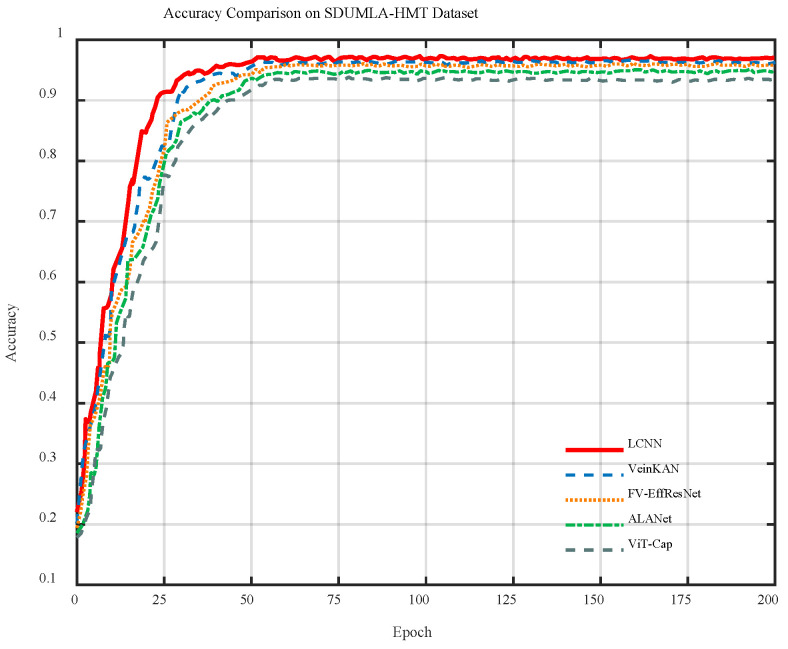
Accuracy curves of different models on the SDUMLA-HMT dataset.

Figure 11 presents the training and validation accuracy curves of each model on the SDUMLA-HMT dataset. As shown, the accuracy of all five models increases rapidly during the first 30 epochs and then gradually stabilizes. Among them, the LCNN demonstrates a faster performance improvement in the early training stage and consistently achieves the highest accuracy throughout the entire training process.

**Figure 12:**
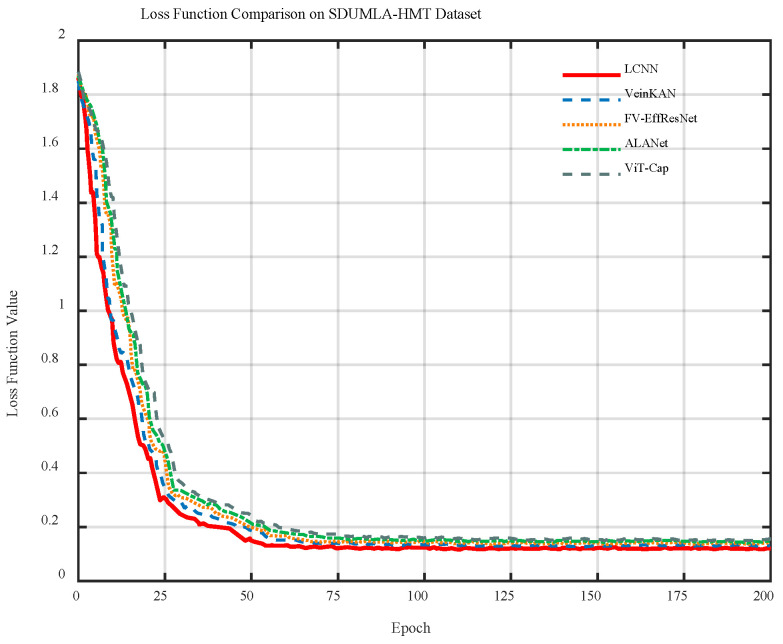
Loss curves of different models on the SDUMLA-HMT dataset.

Figure 12 shows the training loss curves of different models. As observed, the loss values of all models decrease rapidly during the early training stages and become stable around epoch 50. Among them, the LCNN achieves the smoothest and lowest loss trajectory, indicating more effective feature-extraction optimization, faster convergence, and superior training stability compared with the other models.

As shown in Table 4, the proposed LCNN model achieves the highest recognition accuracy of 97.1%, outperforming ViT-Cap by 3.6 percentage points and VeinKAN by 0.9 points. In terms of verification reliability, LCNN attains a low equal error rate (EER) of 0.81%, comparable to the best competing methods. Although FV-EffResNet achieves a slightly lower EER, its accuracy and efficiency are inferior to LCNN. Combined with its compact structure (2.1 M parameters) and low inference latency (3.6 ms), LCNN demonstrates an excellent balance between accuracy, robustness, and computational efficiency.

**Figure 13:**
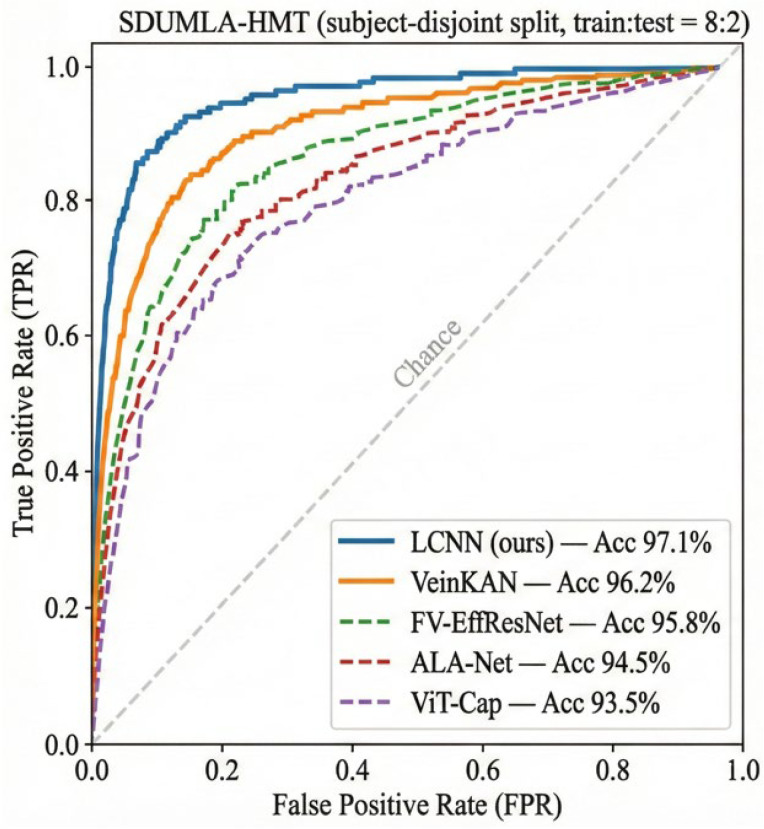
ROC curves of the SDUMLA-HMT finger vein dataset.

The ROC curves on the SDUMLA-HMT dataset are shown in Figure 13. LCNN consistently surpasses all competing models with a higher true positive rate across varying thresholds, which confirms its superior verification robustness.

#### 5.3.2. Experimental Results and Analysis on the Self-Built Lab-Vein Dataset

The performance of the proposed LCNN model was evaluated on the self-built Lab-Vein dataset using several comparative experiments. Five representative CNNs were selected as baselines: VGG-16 [29], GoogLeNet [30], MobileNetV2 [28], EfficientNet [31], and ShuffleNet [32]. VGG-16 is a deep classic network, GoogLeNet adopts Inception modules for multi-scale feature extraction, and MobileNet V2 uses depthwise separable convolutions for lightweight design. EfficientNet applies compound scaling to balance accuracy and complexity, while ShuffleNet employs channel shuffling to improve efficiency. These models cover different architectural philosophies, providing a comprehensive comparison for LCNN in both accuracy and computational efficiency.

**Figure 14:**
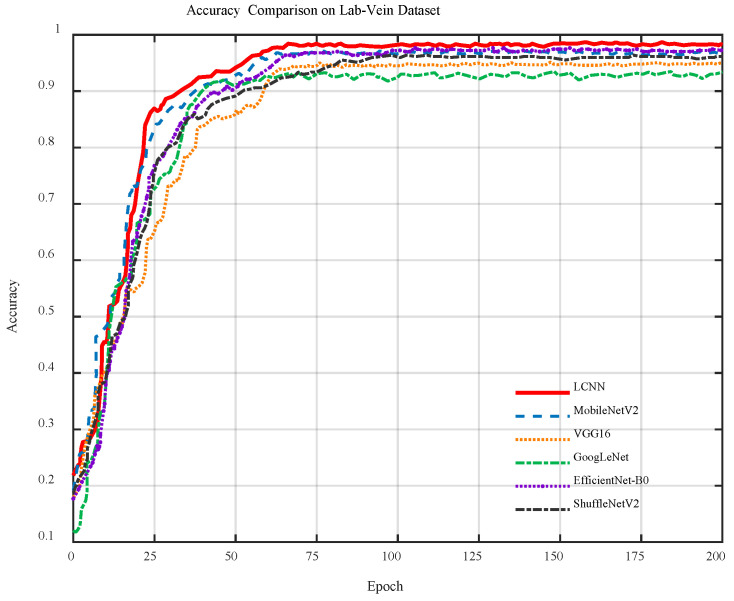
Accuracy curves of different models on the Lab-Vein dataset.

Figure 14 shows the accuracy curves of six models on the Lab-Vein dataset. The accuracy of all models increases rapidly during the first 25 epochs and then gradually converges. LCNN consistently achieves the highest accuracy, followed by EfficientNet-B0 and ShuffleNet-V2, while VGG-16 and GoogLeNet perform slightly worse, demonstrating the strong feature extraction capability of the proposed LCNN.

**Figure 15:**
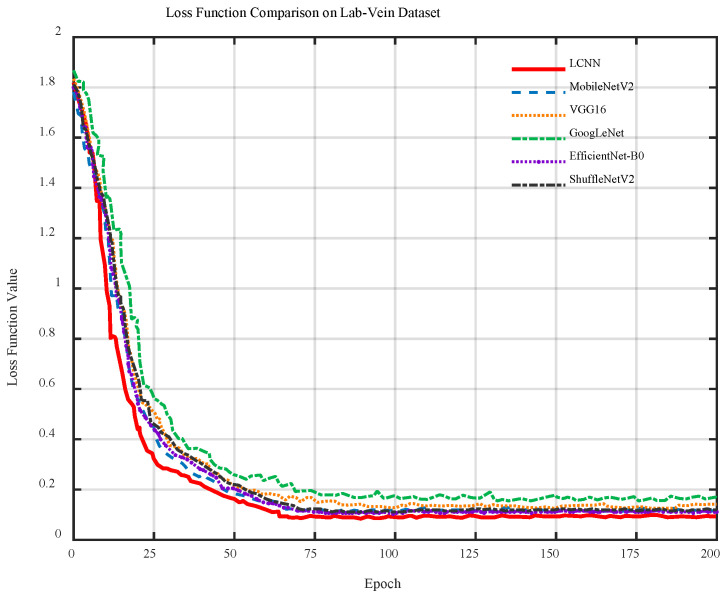
Loss curves of different models on the Lab-Vein dataset.

Figure 15 shows the loss curves of six models on the Lab-Vein dataset. The loss of all models drops rapidly during the first 30 epochs and then gradually stabilizes. LCNN shows the fastest loss reduction and maintains the lowest and most stable values in later stages, indicating faster convergence and more effective feature learning than the other networks.

As shown in Table 5, the proposed LCNN achieves the highest accuracy and F1 score on the Lab-Vein dataset, together with the lowest EER (0.32%), indicating superior verification reliability and classification stability. Compared with MobileNet V2, EfficientNet-B0, and ShuffleNet V2, LCNN exhibits clear advantages, surpassing MobileNet V2 by 1.7 percentage points in F1 score and outperforming VGG-16 and GoogLeNet by 4.0 and 5.5 points, respectively. Moreover, both sensitivity and precision exceed 97%, confirming the well-balanced and robust recognition capability of the proposed model.

Table 6 compares the parameter counts and computational cost (FLOPs) of six networks. Traditional CNNs such as VGG-16 and GoogLeNet are large-scale and computationally intensive, whereas lightweight models, including MobileNet-V2, EfficientNet-B0, ShuffleNet, and LCNN, exhibit substantially reduced complexity. Specifically, LCNN’s parameters and FLOPs are only 1.5% and 1.2% of those of VGG-16, respectively. Compared with MobileNet-V2, LCNN further reduces both metrics by approximately 40%, effectively reducing computational and storage overheads while maintaining high accuracy.

Table 7 summarizes the average inference latency and relative speed-up ratio of the six models. As shown, VGG-16 exhibits the slowest inference, whereas GoogLeNet achieves about 1.9× acceleration. Lightweight architectures, including MobileNet-V2, EfficientNet-B0, ShuffleNet, and LCNN, deliver markedly faster inference speeds. In particular, LCNN reaches an average latency of 12.6 ms, accounting for only 19% of VGG-16’s time (a 5.3× speed-up). Combined with Table 4 and Table 5, these results confirm that LCNN attains high efficiency and maintains competitive accuracy.

**Figure 16:**
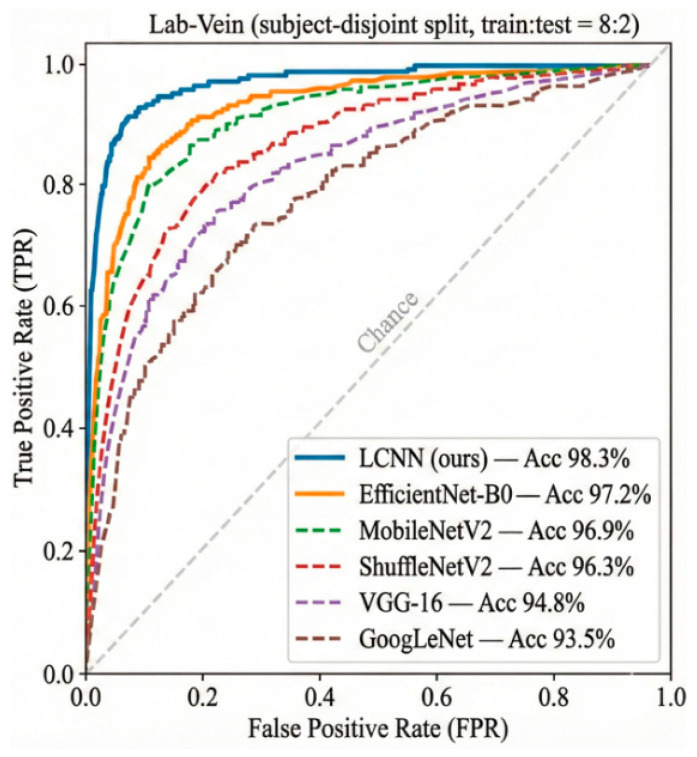
ROC curves of the Lab-Vein finger vein dataset.

The ROC curves on the Lab-Vein dataset are presented in Figure 16. The proposed LCNN exhibits the most favorable operating characteristics among all compared backbones, maintaining a consistently higher true positive rate under the same false positive rate as the decision threshold varies. In particular, LCNN shows a clear advantage in the low-FPR region, indicating stronger separability and more reliable verification performance on the self-collected dataset.

**Figure 17:**
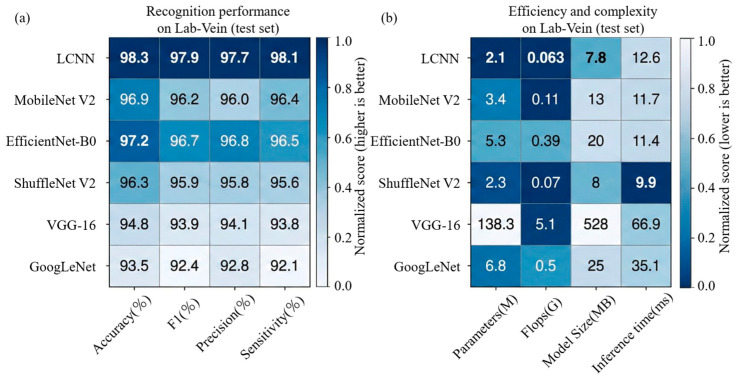
Heatmap comparison of recognition performance and deployment efficiency on the Lab-Vein test set. (**a**) Recognition effectiveness in terms of Accuracy, F1-score, Precision, and Sensitivity (darker shades = higher column-wise normalized scores). (**b**) Efficiency and complexity comparison including Parameters, FLOPs, Model Size, and Inference Time (darker shades = lower computational cost).

Figure 17 presents a dual-panel heatmap comparison on the Lab-Vein test set, summarizing both recognition effectiveness and deployment-related efficiency. Figure 17a reports Accuracy, F1-score, Precision, and Sensitivity, where darker shades denote higher column-wise normalized performance and the overlaid numbers indicate the raw metric values. Figure 17b visualizes Parameters, FLOPs, Model Size, and Inference Time, with the color scale inverted so that lower computational cost appears darker, enabling an intuitive comparison of efficiency among different models.

**Figure 18:**
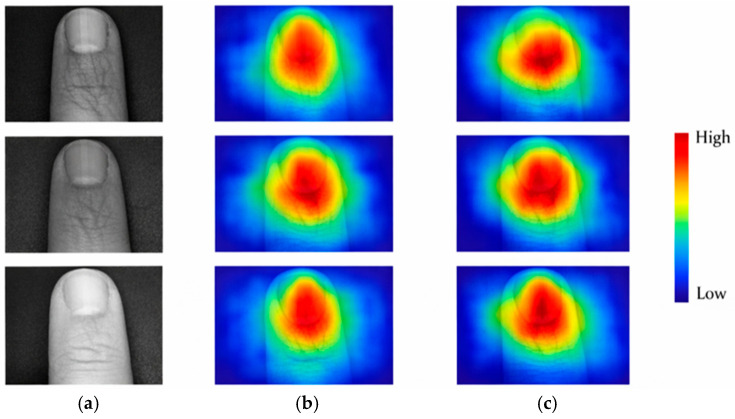
Grad-CAM attention heatmaps for finger-vein recognition models. (**a**) Input images, (**b**) EfficientNet-B0 heatmaps, (**c**) LCNN heatmaps. The proposed LCNN focuses mainly on the central vascular regions, confirming its better feature localization ability.

To visually interpret the model’s behavior, Grad-CAM attention maps were generated, as shown in Figure 18. The proposed LCNN focuses mainly on central vascular regions, whereas EfficientNet-B0 exhibits dispersed attention, confirming LCNN’s stronger capability in capturing discriminative vein features.

#### 5.3.3. Ablation Study

The effectiveness of each key module in the proposed LCNN was examined through an ablation study conducted on the Lab-Vein dataset. Throughout the experiments, the training strategy, data partitioning, and hyperparameter settings were kept consistent with those adopted in the comparative studies. Architectural variations were introduced solely by individually adding or removing specific modules, enabling an analysis of their influence on both recognition accuracy and computational complexity.

(1) Ablation Study Setup

In this study, the baseline network comprises only inverted residual blocks and depthwise-separable convolutions. Building upon this baseline, additional components are progressively introduced: the SE channel-attention module, the Channel Shuffle mechanism, and finally a combined configuration forming the complete LCNN (Full) architecture. For the sake of fair comparison, all experiments were carried out under identical input resolutions, training epochs, and hardware environments for inference-time evaluation.

(2) Recognition Performance Analysis

Table 8 shows the recognition performance of different module combinations. The Baseline network already achieves satisfactory performance on the Lab-Vein dataset. After the introduction of the SE module, both accuracy and F1-score exhibit significant improvement. This result indicates that the SE module enhances the ability of the model to extract discriminative vein features through channel-wise recalibration, thereby suppressing redundant background information and improving overall recognition capability.

When only the Channel Shuffle mechanism is added, model performance also improves to a certain extent. This indicates that Channel Shuffle effectively facilitates cross-channel information interaction under group convolution, thereby enhancing the lightweight network’s ability to capture fine-grained texture information. Furthermore, when both SE and Shuffle modules are introduced simultaneously (+SE + Shuffle), the model achieves better performance than with either module alone. This suggests a complementary relationship between the two mechanisms, where SE enhances feature discrimination and Shuffle promotes information fusion, leading to a synergistic effect in improving recognition performance.

By further incorporating the h-swish6 activation function and the Block-3 multi-scale structure into the complete model (Full), the network achieves its highest recognition accuracy. This result indicates that multi-scale feature extraction enhances the model’s ability to effectively capture structural variations in finger vein textures across varying spatial scales. Meanwhile, the improved nonlinear activation function enhances the model’s representation capability, leading to optimal overall recognition performance.

(3) Model Complexity and Inference Efficiency Analysis

Table 9 summarizes the comparison of parameter count, FLOPs, and inference latency across different ablation configurations. It can be observed that the introduction of the SE module and the multi-scale structure led to a moderate increase in both model parameters and computational cost. However, the overall complexity remains relatively low, and the inference time increases only slightly, still meeting real-time performance requirements. The proposed LCNN achieves consistent performance gains with minimal additional computational overhead when key modules are integrated. This demonstrates a well-balanced trade-off between accuracy and efficiency, making it suitable for deployment in resource-constrained embedded or mobile platforms.

## 6. Conclusions

This study presents an efficient and robust recognition framework for finger-vein identification based on a Lightweight Convolutional Neural Network (LCNN), aiming to overcome the inherent limitations of conventional algorithms that are sensitive to illumination variations, image noise, and excessive model complexity. A multi-stage image preprocessing pipeline is designed to accurately delineate and enhance the vein region, improving texture clarity and ensuring consistent input quality. On this refined representation, the proposed LCNN adopts a structurally optimized architecture that substantially reduces both parameter count and computational cost while maintaining strong feature-representation capability. Consequently, the model achieves high real-time performance and robustness under practical application conditions.

Experimental results on the publicly available SDUMLA-HMT dataset and the self-acquired Lab-Vein dataset yield recognition accuracies of 97.1% and 98.3%, respectively, outperforming several existing lightweight networks. With an average inference time of 12.6 ms per image, the method satisfies real-time requirements for embedded and low-power devices, achieving a balanced trade-off between recognition accuracy and computational efficiency.

Although the proposed LCNN demonstrates promising results, several limitations remain. The current experiments are restricted to static finger-vein images captured under controlled conditions, which may not fully reflect real-world variability. Furthermore, the adaptation of the model to diverse hardware platforms and ultra-low-power environments has not yet been fully explored. Future work will focus on improving cross-device and cross-illumination generalization through adaptive learning and feature-transfer mechanisms, as well as investigating hardware-level optimization and model-compression techniques to facilitate efficient deployment in intelligent terminals and Internet-of-Things applications.

## Figures and Tables

**Table 1:** Performance comparison between classical and DL-based segmentation methods.

Method	Dice (%)	IoU (%)	Inference Time (ms)	Parameters (M)
Otsu-Based (Proposed)	90.5	82.8	8.5	-
U-Net	91.8	84.4	37.2	7.9
MobileNet-U-Net	90.9	83.7	18.6	1.5

Note: The proposed Otsu-based segmentation method is non-learning; therefore, the parameter count is not applicable.

**Table 2:** Structural configuration of the proposed LCNN.

Layer	Input Size	Operator/Block	Output Channels	Stride
Input	96 × 96 × 1	-	-	-
Conv	96 × 96 × 1	3 × 3 Conv	8	2
MaxPool	48 × 48 × 8	3 × 3 MaxPool	8	2
Stage1	12 × 12 × 16	block-1 × 1	16	2
Stage2	12 × 12 × 16	block-2 × 2	32	1
Stage3	12 × 12 × 32	block-3 × 2	64	1
Stage4	12 × 12 × 64	block-4 × 1	64	1
Conv1 × 1	12 × 12 × 64	1 × 1 Conv	128	1
GAP	1 × 1×128	Global AvgPool	128	-
FC	1 × 1×128	Fully connected layer	N	-

**Table 3:** System Configuration of the Experimental Environment.

Environment Configuration	Model Version
CPU processor	Intel Core i7-14650HX @ 2.20 GHz
Internal memory	32GB
GPU graphics card	NVIDIA GeForce RTX 4060
CUDA version	11.1
CUDNN version	8.1
Python version	3.8
PyTorch version	2.2.1
Operating system	Windows 11

**Table 4:** Performance comparison of different models on the SDUMLA-HMT dataset.

Model Name	Years	Accuracy/%	EER (%)	Parameters (M)	Time (ms)
LCNN (Ours)	2026	97.1	0.81	2.1	3.6
ViT-Cap [12]	2022	93.5	4.13	-	-
ALANet [13]	2023	94.5	0.53	2.55	4.32
FV-EffResNet [15]	2024	95.8	0.43	1.32	5.60
VeinKAN [16]	2025	96.2	-	34.81	1.0096

Note: All results in the table represent the final performance of each model on the test set.

**Table 5:** Performance comparison of different models on the Lab-Vein dataset.

Model Name	Accuracy (%)	EER (%)	F1 (%)	Precision (%)	Sensitivity (%)
LCNN(ours)	98.3	0.32	97.9	97.7	98.1
MobileNet V2	96.9	0.68	96.2	96.0	96.4
EfficientNet-B0	97.2	0.55	96.7	96.8	96.5
ShuffleNet V2	96.3	0.92	95.9	95.8	95.6
VGG-16	94.8	1.664	93.9	94.1	93.8
GoogLeNet	93.5	2.13	92.4	92.8	92.1

**Table 6:** Comparison of parameter count, computational cost, and model size across different models.

Model Name	Parameters (M)	FLOPs (G)	Model Size (MB)
LCNN(Ours)	2.1	0.063	7.8
MobileNetV2	3.4	0.11	13
EfficientNet-B0	5.3	0.39	20
ShuffleNet V2	2.3	0.07	8
VGG-16	138.3	5.1	528
GoogLeNet	6.8	0.5	25

**Table 7:** Inference speed and relative acceleration of different models.

Model Name	Time (ms)	Relative Speed-Up (×VGG-16)
LCNN (Ours)	12.6	5.31
MobileNetV2	11.7	5.72
EfficientNet-B0	11.4	6.52
ShuffleNet V2	9.9	7.37
VGG-16	66.9	1.00
GoogLeNet	35.1	1.91

**Table 8:** Ablation study: recognition performance of different configurations.

Model Settings	Precision (%)	F1 (%)
Baseline	97.0	96.6
+SE	97.6	97.2
+Shuffle	97.4	97.0
+SE +Shuffle	98.0	97.6
Full (Ours)	98.3	97.9

**Table 9:** Model complexity comparison of different ablation configurations.

Model Settings	Parameters (M)	FLOPs (G)	Time (ms)
Baseline	1.85	0.057	11.8
+SE	2.03	0.060	12.2
+Shuffle	1.86	0.057	11.9
+SE + Shuffle	2.05	0.060	12.3
Full (Ours)	2.10	0.063	12.6

## Data Availability

The original contributions presented in this study are included in the article. Further inquiries can be directed to the corresponding author.
